# Risk factors associated with IgA vasculitis with nephritis (Henoch–Schönlein purpura nephritis) progressing to unfavorable outcomes: A meta-analysis

**DOI:** 10.1371/journal.pone.0223218

**Published:** 2019-10-01

**Authors:** Dongmei Shi, Han Chan, Xia Yang, Gaofu Zhang, Haiping Yang, Mo Wang, Qiu Li

**Affiliations:** 1 Department of Nephrology, Children’s Hospital, Chongqing Medical University, Chongqing, China; 2 Children’s Hospital of Chongqing Medical University, Yuzhong District, Chongqing, People’s Republic of China; 3 Ministry of Education, Key Laboratory of Child Development and Disorders, Key Laboratory of Pediatrics in Chongqing, Chongqing International Science and Technology Cooperation Center for Child Development and Disorders, Children’s Hospital of Chongqing Medical University, Chongqing, China; University of KwaZulu-Natal, SOUTH AFRICA

## Abstract

**Objective:**

To identify risk factors associated with unfavorable outcomes in children with IgA vasculitis with nephritis (Henoch-Schőnlein purpura nephritis)(IgA-VN)

**Methods:**

PubMed, Embase, and Web of Science databases were searched for studies, published in English through February 2019. The data were extracted to perform pooled analysis, heterogeneity testing, subgroup analysis, sensitivity analysis, and publication bias analysis.

**Results:**

This meta-analysis showed that, older age at onset (WMD 1.77, 95% CI 0.35–3.18, p = 0.014), lower glomerular filtration rate (GFR; WMD -23.93, 95% CI -33.78- -14.09, p<0.0001), initial renal manifestations with nephrotic syndrome (OR 1.74, 95% CI 1.12–2.70, p = 0.013), with nephritic-nephrotic syndrome (OR 4.55, 95% CI 2.89–7.15, p<0.0001) and renal biopsy with crescentic nephritis (International Study of Kidney Disease in Children [ISKDC] grades III-V) (OR 3.85, 95% CI 2.37–6.28, p<0.0001) were significant risk factors associated with poor outcomes in IgA-VN, whereas initial clinical features with hematuria (OR 0.33, 95% CI 0.16–0.69, p = 0.003) and mild proteinuria±hematuria (OR 0.46, 95% CI 0.28–0.75, p<0.0001) were associated with progression to good outcomes. By contrast, gender, hypertension and initial renal manifestations of acute nephritic syndrome were not significantly associated with poor outcomes in IgA-VN.

**Conclusion:**

This meta-analysis showed that older age at onset, lower GFR, initial renal features of nephrotic syndrome and nephritic-nephrotic syndrome and renal biopsy with crescentic nephritis (ISKDC grades III-V) were predictive of poor prognosis in children with IgA-VN.

## Introduction

IgA vasculitis (Henoch-Schönlein purpura)(IgAV) is the most common form of systemic vasculitis in children, with an annual incidence of 10–20 per 100,000 [1]. Although it is generally considered a self-limiting disease in most patients, long-term prognosis depends on the severity of renal involvement. IgA-VN occurs in 30–50% of IgAV patients, mostly in those with a mild form with microscopic hematuria or/and mild proteinuria. However, 1–7% of patients with renal involvement present with more serious damage, manifesting primarily as nephritic or nephrotic syndrome, or even as renal failure, and may progress to end-stage renal disease (ESRD) [2–5]. Identifying risk factors associated with IgA-VN progressing to unfavorable outcomes is crucial to prevent and delay ESRD.

Risk factors associated with the progression of IgA-VN to unfavorable outcomes are unclear. Although pathological changes in the kidney and initial clinical presentation IgA-VN have been reported to be prognostic, other studies show that initial symptoms and histology were not associated with outcomes in patients with IgA-VN [5,6]. Moreover, unfavorable outcomes have been observed in patients with slight renal damage and without crescentic nephritis [7]. This meta-analysis assessed risk factors that may predict progression to unfavorable outcomes in children with IgA-VN.

## Methods

### Literature search strategy

Established methods recommended by the Cochrane Collaboration were used to conduct the meta-analysis [8]. The findings were reported according to the PRISMA (Preferred Reporting Items for Systematic reviews and Meta-Analyses) statement [9]. Laboratory protocols were deposited at protocols.io (http://dx.doi.org/10.17504/protocols.io.6q3hdyn). The PubMed, Embase and Web of Science databases were searched for papers published in English from January 1972 to February 2019, using basic search terms from combined text and Medica Subject Heading (MeSH) terms. These included a MeSH search using the term ‘Purpura, Schoenlein-Henoch’ and a keyword search using the term ‘Henoch-Schönlein purpura’, and terms related to unfavorable outcomes (including MeSH searches using the terms ‘Kidney Failure, Chronic’ and ‘Renal Insufficiency, Chronic’, and keyword searches using the term ‘end stage renal disease’ and ‘chronic renal disease’). This search strategy was adjusted to fit each database (S1 Text). The reference lists of relevant systematic reviews were also checked. There were no restrictions based on publication status. The titles and abstracts of the identified articles were screened, and articles of interest were read in their entirety.

### Study selection

Two reviewers independently screened the titles, abstracts, and full texts of retrieved articles based on pre-specified inclusion and exclusion criteria. Disagreements were resolved by a third reviewer. In the screening process, firstly, subject-irrelevant records were kicked out, through reading of titles and abstracts, then records about prognosis of IgA-VN were preliminary screened through reading full text, finally according to the data extraction requirements, the final records were screened out. Cohort and case-control studies were included, whereas cross-sectional, case reports, review articles, comments, meeting abstracts, genetic association studies, and editorial comments were excluded. Studies were included if they assessed patients diagnosed with IgA-VN at age <18 years; if they included detailed information after the onset of IgA-VN, with a minimum follow-up time of 1 year; and if clinical outcomes was graded according to Meadow’s criteria [10]. Grades of A (normal) and B (minor clinical and urinary abnormalities, including microscopic hematuria or proteinuria <40 mg/m^2^/h, were considered favorable outcomes, whereas grades of C (active renal disease, including hypertension, proteinuria >40 mg/m^2^/h, and increased serum creatinine) and D (uremia/ESRD, including dialysis or renal transplantation) were considered unfavorable outcomes. Patients with IgA nephropathy were excluded.

### Data collection and data extraction

Data were independently extracted by two investigators, with any discrepancies resolved by a third investigator. Data collected included the characteristics of the studies (year of publication, country, and duration of follow-up), the demographic characteristics of the patients (e.g., numbers of patients and age), laboratory predictors, renal manifestations and renal histopathology at onset.

Patients were subdivided into five classes according to the renal manifestations at onset of IgA-VN [10]: 1) hematuria, with micro- or macroscopic hematuria defined as (>5 red blood cells (RBCs)/HP and >20 RBCs/10^6^/L, respectively); 2) mild proteinuria ± hematuria, with persistent mild proteinuria defined as <1 g/L or urine albumin/urine creatinine ratio (Ua/c) <200 mg/mmol, and/or hematuria; 3) acute nephritic syndrome, defined as moderate proteinuria (Ua/c ≥ 200–400 mg/mmol), hematuria, increased serum creatinine and/or hypertension [11]; 4) nephrotic syndrome, defined as urinary albumin excretion >40 mg/h/m2 or Ua/c > 400 mg/mmol, serum albumin <25 g/L and/or edema; and 5) mixed nephritic-nephrotic syndrome. Biopsy findings in patients were graded according to the criteria of the International Study of Kidney Disease in Children (ISKDC) [12]: (I) minimal glomerular abnormalities, (II) mesangial proliferation (MP), (III) MP with <50% crescents, (IV), MP with 50–75% crescents, (V) MP with >75% crescents, and (VI) membranoproliferative-like lesions, with ISKDC grades III–V defined as crescentic nephritis. All patients showed predominant IgA deposition on immunofluorescence examination.

### Quality assessments of the studies

These papers are reported following the STROBE (Strengthening the Reporting of Observational Studies in Epidemiology) statement [13]. Study quality was assessed using three main categories of the Newcastle-Ottawa scale (NOS; (http://www.ohri.ca/programs/clinical_epidemiology/oxford.asp): study selection, comparability of groups, and determination of outcomes [14].

### Data synthesis and Statistical analysis

In studies, mean and standard deviation (SD) were not directly presented; rather, they were calculated from EXCEL spreadsheets, as were the combined means and SDs of two groups. Data expressed in two forms in different studies, such as means and SDs or odds ratios (ORs) and 95% confidence intervals (CIs), were pooled by converting all data to ORs and 95% CIs, presented as means and SDs or as medians and quartiles, were not pooled because of large heterogeneity. If a study had two follow-up outcomes, data from the most recent follow-up were included in the meta-analysis.

Aggregates were pooled used the generic inverse variance meta-analysis method. Effect measures of interest were reported as ORs and 95% CIs. If there was significant heterogeneity, a random-effects model was used, or else fixed-effects model. Heterogeneity was tested using the I^2^ test, with I^2^> 50% or P-value <0.1 considered significant. Statistical significance defined as a 2-sided p-value < 0.05. Heterogeneity was assessed by subgroup analyses of follow-up durations(followed up for <5 or >5 years), ethnicity (Europe or Asia), date of publication (before 2000 or after 2000) and study quality across studies. Sensitivity analyses were conducted by removing each individual study from the overall analysis [15]. If fewer than 10 studies were included, publication bias was not evaluated. All statistical analyses were performed using Stata 14.0 software (Stata Corp, College Station, TX, USA) [16].

## Results

### Study selection and characteristics

**Fig 1:**
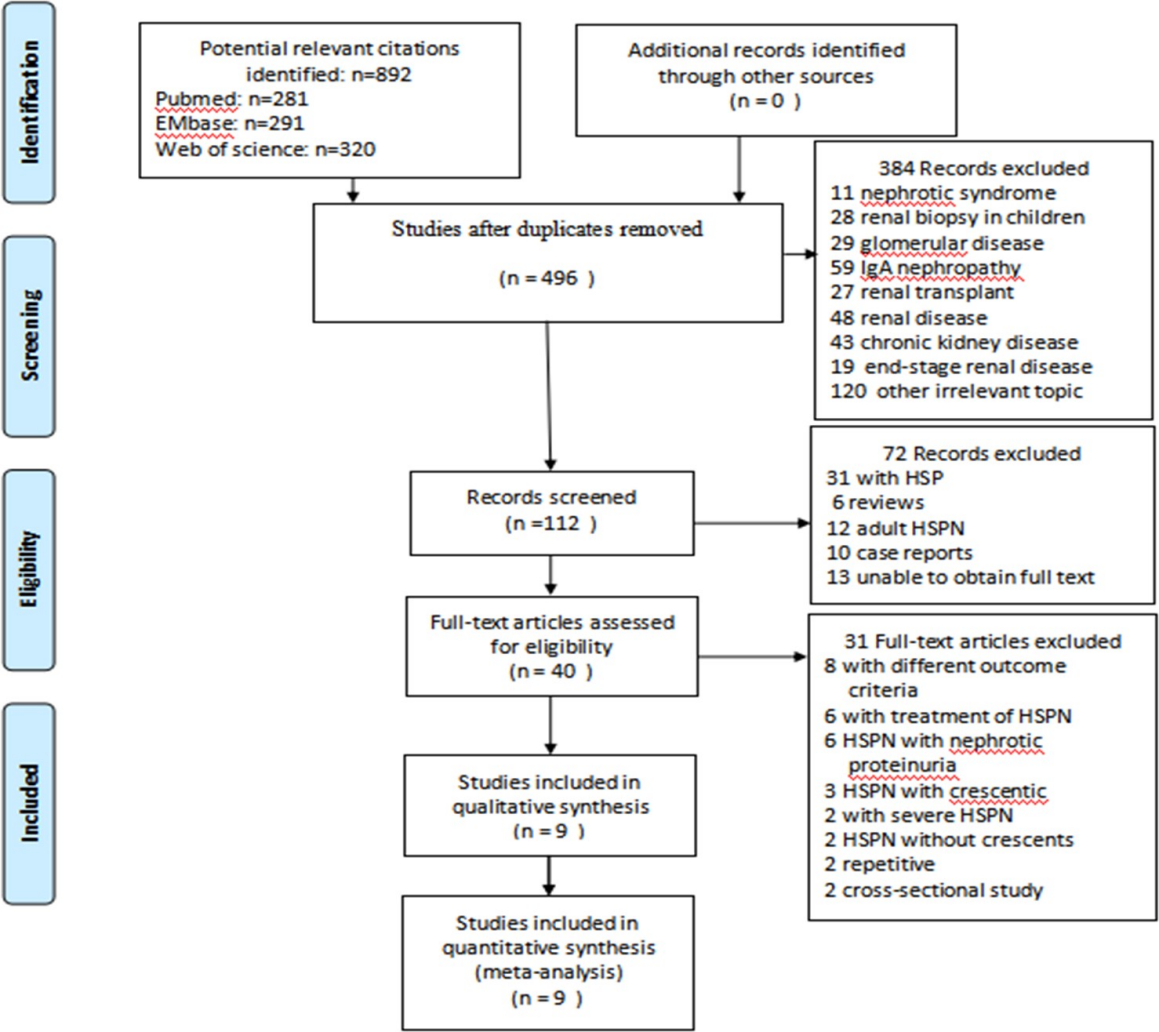
Flowchart of selection process for eligible studies.

Initial screening identified 892 publications (Fig 1). Of these, only 9 case-control studies satisfied our inclusion criteria and were included in the meta-analysis (Table 1). These studies were published from January 1972 to February 2019. We obtained the full text and data of these studies. Of these 9 studies, two each in Finland and Turkey; one each in United Kingdom, Sweden, Japan, Poland and Germany. Thus, six studies were performed in Europe, three in Asia. The 9 studies included 969 patients with HSPN, with 160 experiencing unfavorable outcomes. The follow-up period ranged from 1.0 to 23.4 years, with the follow-up period in six studies being more than 5 years.

**Table 1:** Basic characteristics of included studies.

Studies	Year	Setting	Ethnicity	Mean follow-up, yr	unfavorable/all, n	numbers of renal biopsies/all, n
Koskela[17]	2017	Finland	Europe	7.3	8/53	53/53
Edström[18]	2010	Sweden	Europe	5.2	20/78	59/78
Soylemezoglu[6]	2009	Turkey	Asia	2.7	51/443	179/443
Sevgi[19]	2007	Turkey	Asia	4.3	8/82	82/82
Kawasaki[20]	2003	Japan	Asia	8.7	20/114	114/114
Schärer[21]	1999	Germany	Europe	4.3	21/64	64/64
Goldstein[22]	1992	United Kingdom	Europe	23.4	22/78	70/78
Koskimies[23]	1981	Finland	Europe	7.2	3/29	29/29
Wozniak[24]	2013	Poland	Europe	13.5	7/28	28/28

### Quality of evidence

The quality of the evidence in 9 case-control studies was assessed using the NOS scale (Table 2). Seven studies were judged to be of high relative quality and two of medium quality. The control groups in the selected studies were not community-based. Because these studies were published between 1981 and 2017, there may have been a bias towards different treatment strategies. In addition, patients in three studies were followed up for less than 5 years, which may introduced bias.

**Table 2:** Newcastle-Ottawa quality assessment scale (case-control) for 9 studies^a^ included in this meta-analysis.

		1	2	3	4	5	6	7	8	9
Was the case definition adequate	a. Yes, with independent validation*; b. yes, e.g., record linkage or based on self-reports; c. no description	*	*	*	*	*	*	*	*	**
Representativeness of the cases	a. Consecutive or obviously representative series of cases*; b. potential for selection biases or not stated	*	*	*	*	*	*	*	*	*
Selection of controls	a. Community controls*; b. hospital controls; c. no description									
Definition of controls	a. No history of disease (endpoint)*; b. no description of source	*	*	*	*	*	*	*	*	*
Comparability	a. Study controls for_ _ _ _(selecting the most important factor)*; b. study controls for any additional factor*	*	*		*	*	*	*	*	
Ascertainment of exposure	a. Secure records (e.g., surgical records)*; b. structured interview blinded to case/control status; interview not blinded to case/control status*; d. written self-report or medical record only; e. no description	*	*	*	*	*	*	*	*	*
Ascertainment for cases & controls	a. Yes* b. No	*	*	*	*	*	*	*	*	*
Non-response rate	a. Same rate for both groups*; b. non-respondents described; c. rate different and no designation	*	*	*	*	*	*	*	*	*
score		7	7	6	7	7	7	7	7	6

^a^ Studies: 1, Koskela et al. 2017; 2, Edström et al. 2010; 3, Soylemezoglu et al. 2009; 4, Sevgi et al. 2007; 5, Kawasaki et al. 2003; 6, Schärer et al. 1999; 7, Goldstein et al. 1992; 8, Koskimies et al. 1981; 9, Wozniak et al. 2013.

*Scored points.

Studies were assorted according to the prognosis (favorable or unfavorable) of patients with IgA-VN. The control group in each study was selected from the same population as the case group, with all control subjects showing favorable outcomes after follow-up. Control subjects were selected independently by exposure status and without special clinical features. Cases and controls were followed up for a similar length of time and had similar rates of non-response. To ensure that studies were efficient and valid, the selection and restriction of enrolled patients was evaluated.

### Results of meta-analysis

#### Initial characteristics and laboratory findings

**Fig 2:**
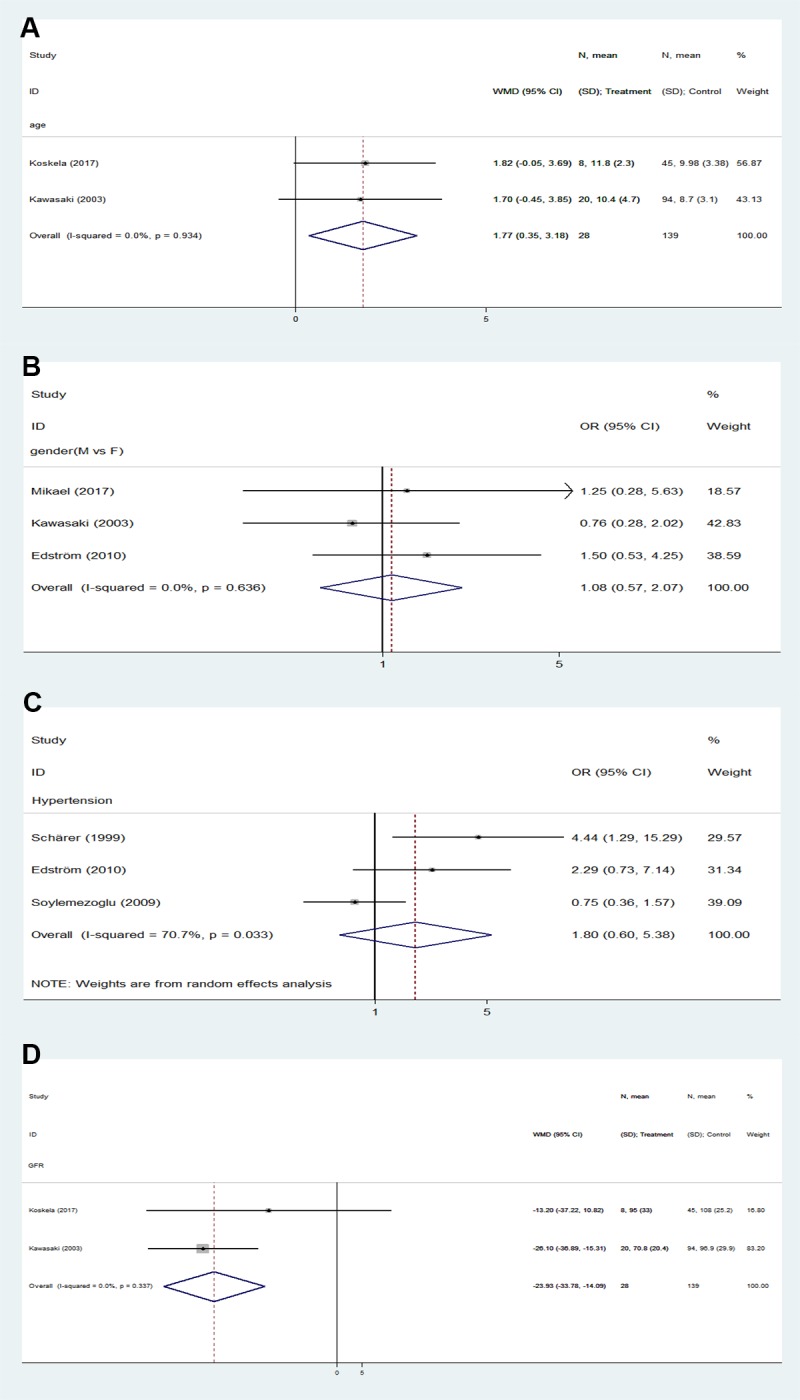
Forest plots of OR/WMD estimates for the following. (A) age. (B) gender (male vs. female). (C) hypertension. (D) level of GFR.

The associations of baseline demographic and clinical characteristics (e.g., age, gender, and hypertension) and laboratory predictors (GFR, serum creatinine, plasma albumin, and level of proteinuria) of study subjects with prognosis in patients with IgA-VN were analyzed to assess risk factors for unfavorable outcomes. Older age (weighted mean difference [WMD] 1.77, 95% CI 0.35–3.18, p = 0.014) and lower GFR level (WMD -23.93, 95% CI -33.78- -14.09, p<0.0001) at onset were risk factors for unfavorable outcomes (Fig 2). By contrast, sex (male vs. female; OR 1.08, 95% CI 0.57–2.07, p = 0.808) and hypertension (OR 1.80, 95% CI 0.60–5.38, p = 0.292) at onset did not significantly affect patient outcomes. Unfortunately, the absence of data or differences in reporting prevented a determination of the effects of serum creatinine, plasma albumin and level of proteinuria on patient outcomes.

#### Initial renal manifestations

**Fig 3:**
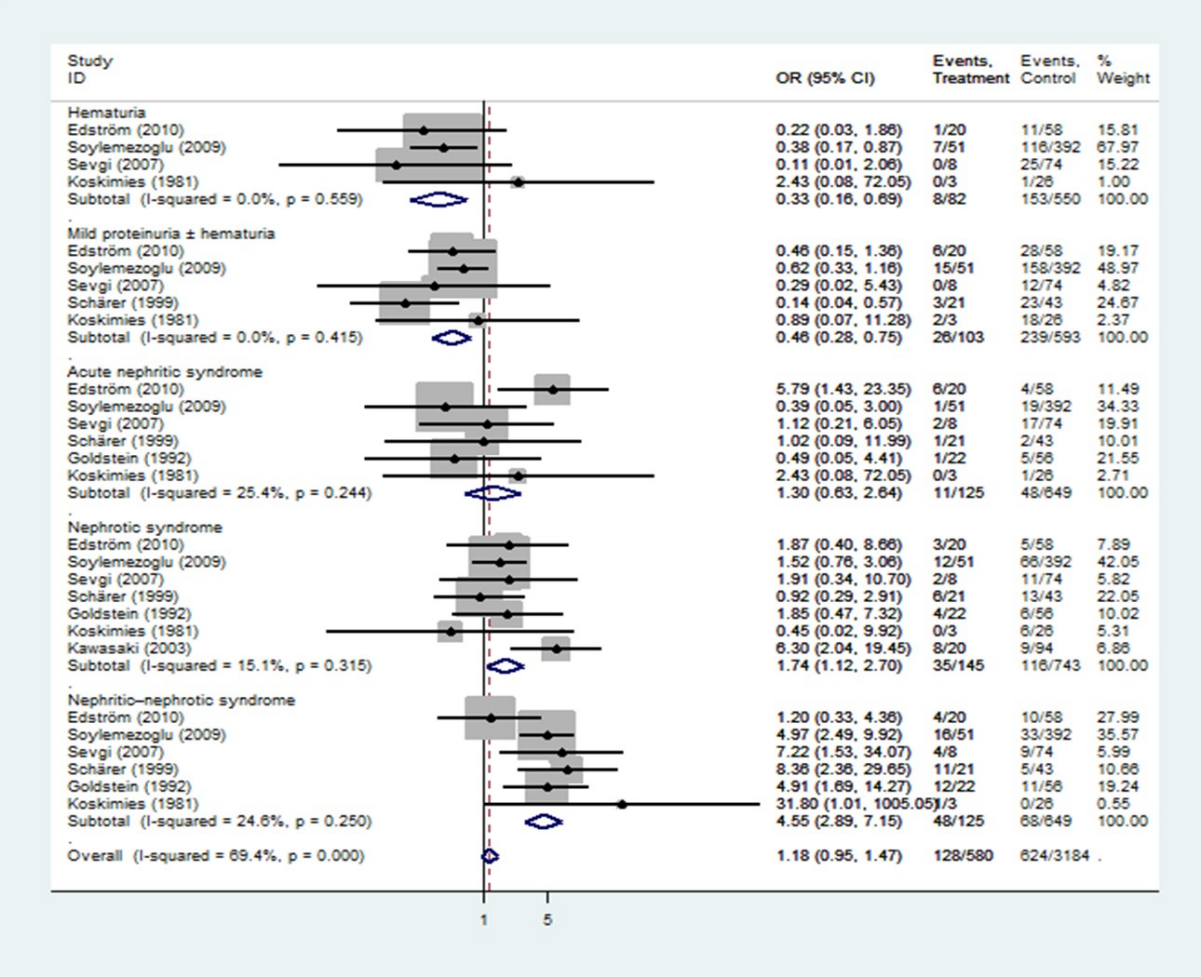
Forest plots of OR estimates for initial clinical features: hematuria, mild proteinuria±hematuria, acute nephritic syndrome, nephrotic syndrome, and nephritic-nephrotic syndrome.

Patients with HSPN were subdivided into five classes based on renal manifestations at onset: hematuria, mild proteinuria±hematuria, acute nephritic syndrome, nephritic syndrome, nephritic-nephrotic syndrome. We found that initial renal features with nephrotic syndrome (OR 1.74, 95% CI 1.12–2.70, p = 0.013) and nephritic-nephrotic syndrome (OR 4.55, 95% CI 2.89–7.15, p<0.0001) at onset was associated with poor prognosis, whereas hematuria (OR 0.33, 95% CI 0.16–0.69, p = 0.003) and mild proteinuria±hematuria (OR 0.46, 95% CI 0.28–0.75, p<0.0001) were associated with good outcomes (Fig 3).

#### Initial renal biopsy

**Fig 4:**
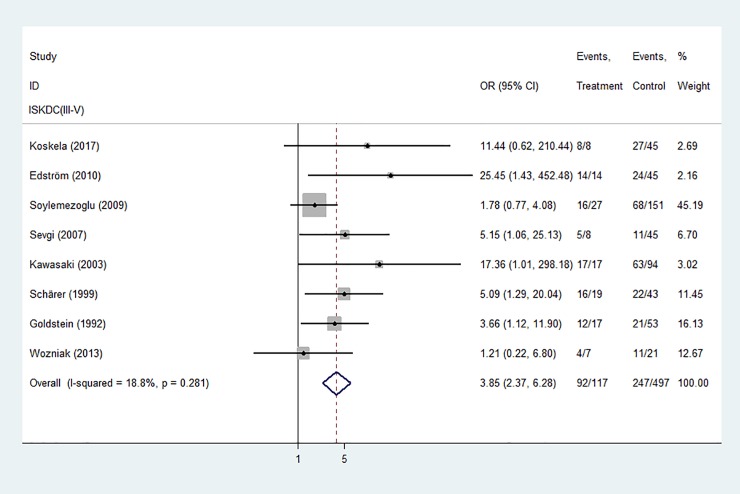
Forest plots of OR estimates for initial renal biopsy: Crescentic nephritis (ISKDC grades III-V).

Biopsy findings in children with HSPN were graded according to the criteria of the International Study of Kidney Disease in Children (ISKDC), with ISKDC grades III-V defined as crescentic nephritis. Initial renal biopsy showing crescentic nephritis (ISKDC grades III-V) was significantly associated with a poor prognosis is children with HSPN (Fig 4).

The associations of theses factors, including baseline demographic, initial characteristics, GFR, initial renal features, and initial renal biopsy with crescentic nephritis (ISKDC grades III-V), with prognosis in patients with HSPN were analyzed to assess risk factors for unfavorable outcomes. The results of this meta-analysis are summarized in Table 3. In conclusion, older age, lower GFR, nephrotic syndrome, nephritic-nephrotic syndrome, and crescentic nephritis were associated with a poor prognosis.

**Table 3:** Results of meta-analysis.

Risk factors	Result
Age	WMD = 1.77,95%CI(0.35,3.18), z = 2.45,p = 0.014
Gender (M vs. F)	OR = 1.08,95%CI(0.57,2.07), z = 0.24,p = 0.808
Hypertension	OR = 1.80,95%CI(0.60,5.38), z = 1.05,p = 0.292
GFR	WMD = -23.93,95%CI(-33.78- -14.09), z = 4.77,p<0.0001
Hematuria	OR = 0.33,95%CI(0.16,0.69),z = 2.99,p = 0.003
Mild proteinuria ± hematuria	OR = 0.46,95%CI(0.28,0.75),z = 3.14,p<0.0001
Acute nephritic syndrome	OR = 1.30,95%CI(0.63,2.64),z = 0.71,p = 0.477
Nephrotic syndrome	OR = 1.74,95%CI(1.12,2.70),z = 2.48,p = 0.013
Nephritic-nephrotic syndrome	OR = 4.55,95%CI(2.89,7.15),z = 6.56,p<0.0001
ISKDC(III–V)	OR = 3.85,95%CI(2.37,6.28),z = 5.42,p<0.0001

WMD: weighted mean difference; OR: odds ratio; CI: confidence interval; GFR: glomerular filtration rate; ISKDC: International Study of Kidney Disease in Children.

#### Subgroup and sensitivity analysis

**Fig 5:**
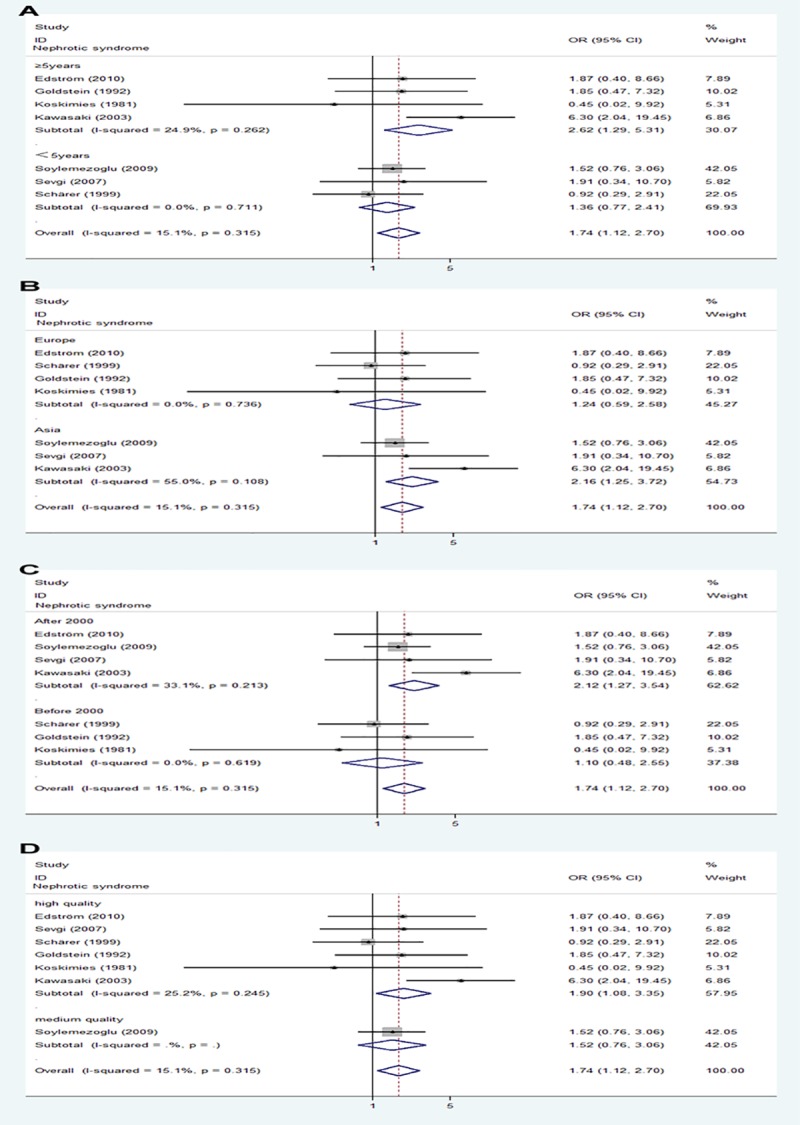
Subgroup analysis of in renal features with nephrotic syndrome. (A) follow-up duration. (B) ethnicity. (C)date of publication. (D) study quality.

**Fig 6:**
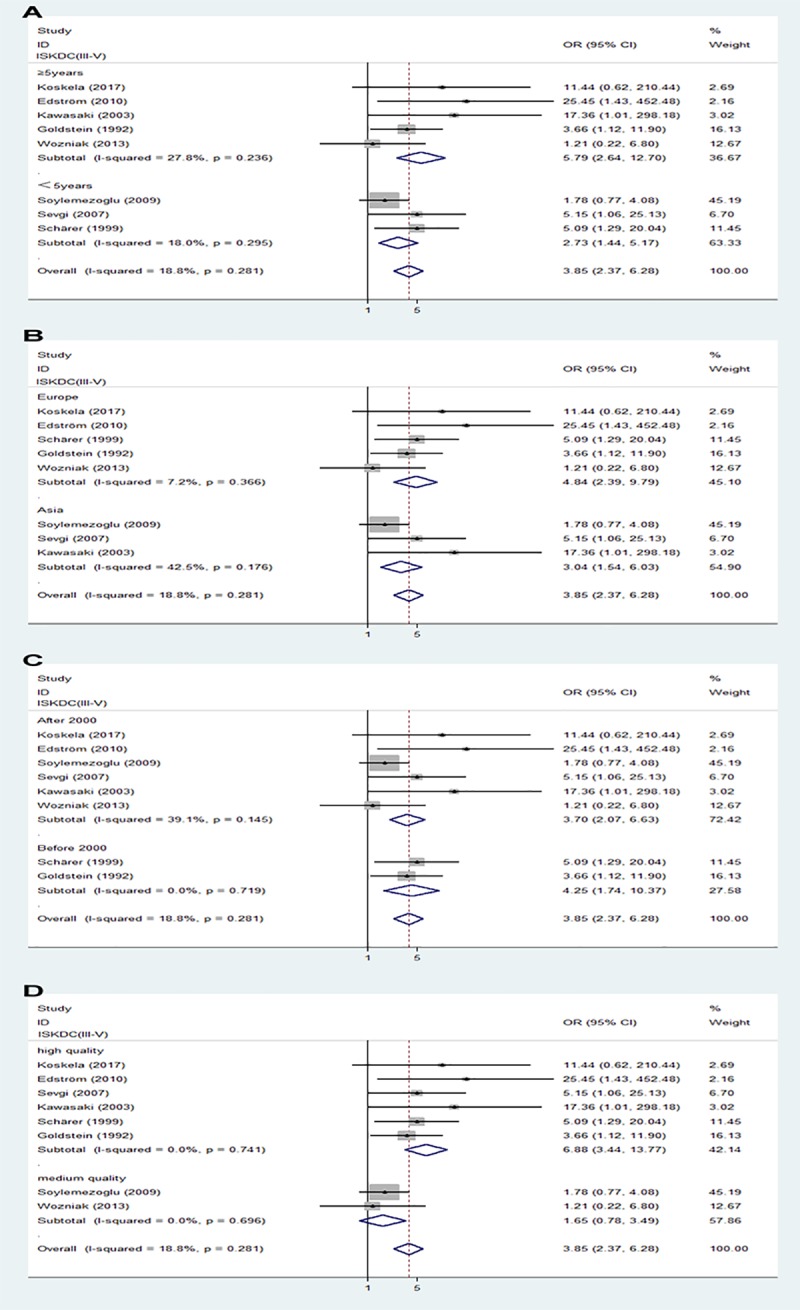
Subgroup analysis of in initial renal biopsy with crescentic nephritis (ISKDC grades III-V). (A) follow-up duration. (B) ethnicity. (C) date of publication. (D) study quality. Sensitivity analysis showed that no individual study significantly altered the effects of nephrotic syndrome, nephritic-nephrotic syndrome and initial renal biopsy with crescentic nephritis on patient prognosis (Fig 7).

Subgroup analysis of follow-up duration (followed up for <5 or >5 years), ethnicity (Europe or Asia), date of publication (before 2000 or after 2000) and study quality showed differences in renal features with nephrotic syndrome. In those groups, followed up for >5 years, patients in Asia, publication after 2000, high quality studies, patients with initial renal features with nephrotic syndrome were associated with progression to poor outcomes (Fig 5). Meanwhile subgroup analysis of study quality showed differences in crescentic nephritis (ISKDC grades III-V), in that high quality studies, crescentic nephritis (ISKDC grades III-V) was associated with progression to poor outcomes (Fig 6). By contrast, there were no differences in prognosis in follow-up duration, ethnicity, or date of publication, who had crescentic nephritis (ISKDC grades III-V) at diagnosis (Fig 6). In addition, prognosis was not affected by follow-up duration, ethnicity, date of publication, or study quality, in who had nephritic-nephrotic syndrome at diagnosis (S1 Fig).

**Fig 7:**
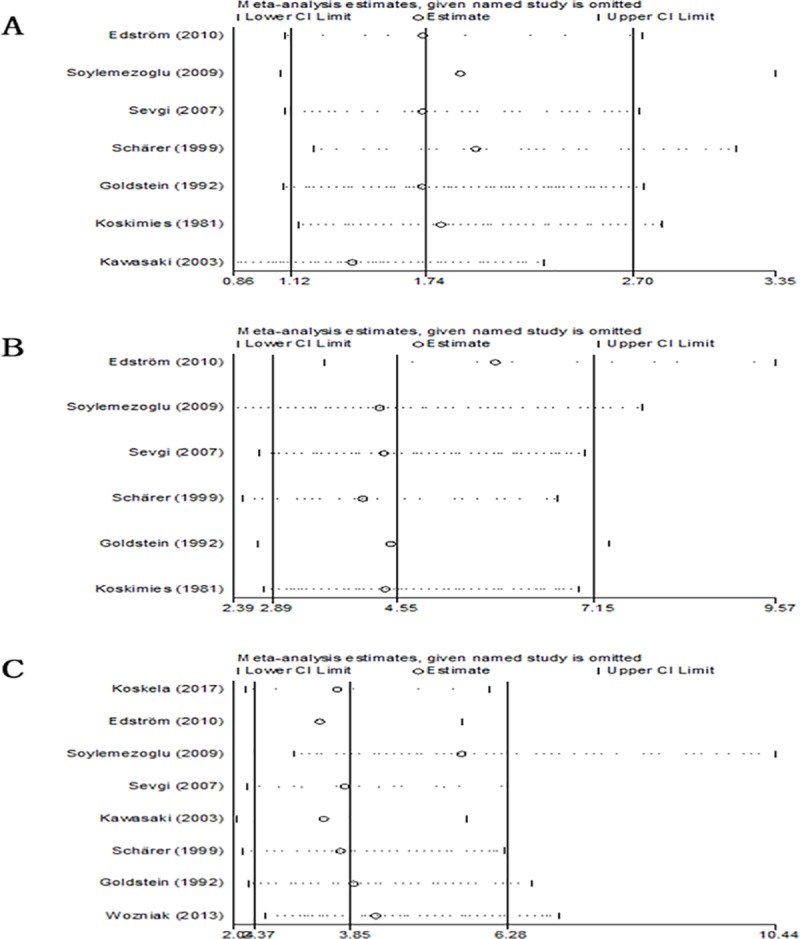
Sensitivity analysis. (A) nephrotic syndrome. (B) nephritic-nephrotic syndrome. (C) initial renal biopsy with crescentic nephritison.

#### Publication bias

In this meta-analysis, publication bias was not evaluated for fewer than 10 studies were included.

## Discussion

Although the long-term prognosis of children with IgA-VN and the risk factors for unfavorable outcomes have been analyzed, no systematic review and meta-analysis has analyzed factors associated with long-term unfavorable outcomes in children with IgA-VN. This meta-analysis analyzed several categories of risk factors, including demographic features (e.g., age and gender), clinical features (e.g., hypertension), initial renal manifestations (e.g., hematuria, mild proteinuria±hematuria, acute nephritic syndrome, nephrotic syndrome, and nephritic-nephrotic syndrome), GFR, and renal histopathology ISKDC class.

Older age was associated with an unfavorable prognosis in children with IgA-VN [18,25,26]. Consistent with these findings, the present meta-analysis included two studies showing that older age is a risk factor for unfavorable outcomes in patients with IgA-VN. However, two studies, without merging for difference type of data type or without detail data provided, found that older age was not a risk factor for poor outcomes in patients with IgA-VN [12,19,20]. These inconsistent conclusions suggest the need for further studies assessing the relationship between age and unfavorable prognosis in IgA-VN patients.

In contrast to a previous meta-analysis, which suggested that boys are at higher risk for renal involvement than girls [27], our meta-analysis based on three studies showed no association between gender and outcome. Moreover, two other studies [12,19] reported no association between gender and long-term outcomes of IgA-VN.

Patients were divided into five groups based on renal manifestations at onset: hematuria, mild proteinuria±hematuria, acute nephritic syndrome, nephrotic syndrome, and nephritic-nephrotic syndrome[10]. Most identified studies showed an association between renal features at onset (nephritic syndrome) and poor prognosis [28–30]. A multivariate logistic regression analysis showed that a nephrotic state lasting longer than 3 months had a significant effect on unfavorable outcomes [25]. As the same, our analysis showed that nephrotic syndrome and nephritic-nephrotic syndrome at diagnosis were a significant independent predictor of poor prognosis, whereas acute nephritic syndrome was not. Conversely initial hematuria and mild proteinuria±hematuria were associated with more favorable outcomes.

Our meta-analysis also showed that hypertension was unrelated to poor outcomes. Three studies reported that recurrence of non-renal symptoms did not correlate with poor outcomes [12,21,31], whereas one study [26] indicated that ≥4 purpura relapses was prognostic of poor outcomes in patients with HSPN. Additional studies are required to determine whether purpura relapses are prognostic of poor outcomes in patients with IgA-VN.

Laboratory findings showed that a lower level of GFR was a risk factor for progressing to unfavorable outcomes in IgA-VN patients. Also, Edström reported [18], at the 1-year follow-up (patients were studied 1 year from onset), patients with a poor outcome were lower GFR than patients with a good outcome. While Xu et al. [28] noted that GFR at onset was not signifcant variables for clinical remission in IgA-VN patients. Additional studies were needed to explored GFR at onset or follow-up with prognostic of IgA-VN. Poteinuria was an important indicator of the severity of kidney damage in the clinic. In this meta-analysis, three studies reported that proteinuria at onset was correlate with poor outcomes [17,18,20], unfortunately, data about proteinuria was not pooled because of the different data presentation and measurement method proteinuria. Follow-up proteinuria was also an important indicator in prognostic predictor in IgA-VN. Edström reported [18], at the 1-year follow-up (patients were studied 1 year from onset), patients with a poor outcome had a higher Ua/c (urine albumin/urine creatinine ratio). Another study showed that [5], mean proteinuria during follow-up was a independent prognostic predictor in IgA-VN in children and adults. So additional studies were required to explored proteinuria at onset or follow-up with prognostic of IgA-VN. Because of differences in data presentation, we were unable to merge data about plasma creatinine and albumin concentrations. Although IgA-VN is an immune-related disease, no study to date has assessed the correlation between immune-related indicators and patient prognosis, indicating a need for further research.

A kidney biopsy remains a gold standard for evaluating the severity of IgA-VN and its associated prognosis. Many previous studies showed that patients with a high ISKDC grades III-V on first biopsy may have unfavorable long-term outcomes. Our analysis showed that initial renal biopsy with crescentic nephritis (ISKDC grades III-V) was a significant independent predictor of an unfavorable outcome. As the same, initial renal biopsy with ISKDC grades IV-V and ISKDC grades V were associated with unfavorable outcome (S2 Fig), indicating that the more percentage of crescents, more inclined to prognosis to unfavorable outcome in patients with IgA-VN. However patients with low ISKDC grades (I and II) may have poor outcomes [22,32,33]. Because ISKDC grades are based mainly on the presence and number of affected glomeruli, separate evaluation of activity and chronicity components is important. Only two studies reported that acute and chronic changes were more frequent in patients with unfavorable than favorable prognosis [17,20]. Differences in scoring systems for acute and chronic changes in renal histopathology prevented their combined analysis. Use of the Oxford classification of IgA nephropathy, which includes five renal features: mesangial hypercellularity (M), endocapillary proliferation (E), segmental glomerulosclerosis (S), tubular atrophy/interstitial fbrosis (T), and cellular/fbrocellular crescents (C) [28], to assess the degree of kidney damage indicated that S was strongly associated with the primary outcome, whereas T was associated with proteinuria remission and clinical remission. In our analysis, crescentic nephritis on renal biopsy was a predictor of unfavorable outcomes. Further studies are needed to explore the relationship between renal histopathology and unfavorable prognosis in IgA-VN patients. A sensitive scoring system based on histopathological changes at onset or follow-up may help predict poor outcomes in patients with IgA-VN.Following limitations should be considered in this meta-analysis. First, this analysis was based only on cohort and case-control studies, and randomized controlled studies are needed to explored the above risk factors are relied on as causal factors associated with IgA vasculitis with nephritis (Henoch-Schönlein purpura nephritis) progressing to unfavorable outcomes. Second, some of the risk factors studied, such as age, hypertension, proteinuria and GFR, were assessed in few publications, and differences in data presentation or details prevented a more robust meta-analyses of these factors, and some more recent articles could not be included in the analysis. Third, clinical and pathology features at baseline only are considered, without taking into account the treatment performed in the evaluation of the risk of progression over long term follow-up, may have been confounding factors in the included studies. Fourth, there were selection bias because of a small part literatures were exclusion, in that papers did not use Meadow's criteria. The wide range in publication dates, follow-up durations and ethnic populations, coupled with the development of new treatment technologies, may have been confounding factors in the included studies. Finally, few laboratory indicators were included, and it was impossible to comprehensively review all possible risk factors.

## Conclusions

In conclusion, our results provide a detailed overview of factors associated with prognosis in children with IgA-VN. Older age, lower GFR, nephrotic syndrome, nephritic-nephrotic syndrome, and crescentic nephritis were associated with a poor prognosis. Children who have one or more of these risk factors should be monitored periodically, and early treatment of these patients is warranted. A sensitive scoring system based on these risk factors at onset, including detailed age stratification, more laboratory indicators, follow-up proteinuria, renal biopsy at onset or follow-up and so on, may better predict poor outcomes in children with IgA-VN.

## Supporting information

S1 Appendix PRISMA 2009 checklist. (DOC)

S1 Fig Subgroup analysis of in renal features with nephritic-nephrotic syndrome: (A) follow-up duration; (B) ethnicity; (C) date of publication; (D) study quality.
(TIF)

S2 Fig Forest plots of OR estimates for initial renal biopsy: (A) ISKDC grades IV-V; (B) ISKDC grades V.
(TIF)

S1 Text Search strategy for each database. (DOCX)
