# Supplementary material for: Risk factors associated with IgA vasculitis with nephritis (Henoch–Schönlein purpura nephritis) progressing to unfavorable outcomes: A meta-analysis
Source: PLoS One. 2019 Oct 1;14(10):e0223218. doi: 10.1371/journal.pone.0223218 (PMC6772070; doi:10.1371/journal.pone.0223218)
Supplement: S1 Appendix — (DOC) [file pone.0223218.s001.doc]

| **Section/topic** | **#** | **Checklist item** | **Reported on page #** |
| --- | --- | --- | --- |
| **TITLE** | | |  |
| Title | 1 | Risk factors associated with Henoch–Schönlein purpura nephritis progressing to unfavorable outcomes: a meta-analysis | 1 |
| **ABSTRACT** | | |  |
| Structured summary | 2 | Objective  To identify risk factors associated with unfavorable outcomes in children with Henoch-Schőnlein purpura nephritis (HSPN)  Methods  PubMed, Embase, and Web of Science databases were searched for studies, published in English through February 2019. The data were extracted to perform pooled analysis, heterogeneity testing, subgroup analysis, sensitivity analysis, and publication bias analysis.  Results  This meta-analysis showed that, older age at onset (WMD 1.77, 95% CI 0.35-3.18, p=0.014), lower glomerular filtration rate (GFR; WMD -23.93, 95% CI -33.78- -14.09, p=0.000), initial renal manifestations with nephrotic syndrome (OR 1.74, 95% CI 1.12-2.70, p=0.013), with nephritic-nephrotic syndrome (OR 4.55, 95% CI 2.89-7.15, p=0.000) and renal biopsy with crescentic nephritis (International Study of Kidney Disease in Children [ISKDC] grades III-V) (OR 3.85, 95% CI 2.37-6.28, p=0.000) were significant risk factors associated with poor outcomes in HSPN, whereas initial clinical features with hematuria (OR 0.33, 95% CI 0.16-0.69 , p=0.003) and mild proteinuria±hematuria (OR 0.46, 95% CI 0.28-0.75, p=0.000) were associated with progression to good outcomes. By contrast, gender, hypertension and initial renal manifestations of acute nephritic syndrome were not significantly associated with poor outcomes in HSPN.  Conclusion  This meta-analysis showed that older age at onset, lower GFR, initial renal features of nephrotic syndrome and nephritic-nephrotic syndrome and renal biopsy with crescentic nephritis (ISKDC grades III-V) were predictive of poor prognosis in children with HSPN. | 2-3 |
| **INTRODUCTION** | | |  |
| Rationale | 3 | HSP nephritis (HSPN) occurs in 30–50% of HSP patients, mostly in those with a mild form with microscopic hematuria or/and mild proteinuria. However, 1-7% of patients with renal involvement present with more serious damage, manifesting primarily as nephritic or nephrotic syndrome, or even as renal failure, and may progress to end-stage renal disease (ESRD). | 3-5 |
| Objectives | 4 | Participants: patients diagnosed with HSPN at age <18 years, with unfavorable outcomes according to Meadow’s criteria.  Interventions : None  Comparisons: patients with favorable outcomes according to Meadow’s criteria.  Outcomes: Epidemiologic and clinical features and some abnormal laboratory and initial renal biopsy findings.  Study design: Cohort studies or case-control studies | 5-7 |
| **METHODS** | | |  |
| Protocol and registration | 5 | None |  |
| Eligibility criteria | 6 | patients diagnosed with HSPN at age <18 years; if they included detailed information after the onset of HSPN, with a minimum follow-up time of 1 year; and if clinical outcomes was graded according to Meadow’s criteria . Grades of A (normal) and B (minor clinical and urinary abnormalities, including microscopic hematuria or proteinuria <40 mg/m2/h, were considered favorable outcomes, whereas grades of C (active renal disease, including hypertension, proteinuria >40 mg/m2/h, and increased serum creatinine) and D (uremia/ESRD, including dialysis or renal transplantation) were considered unfavorable outcomes. Patients with IgA nephropathy were excluded. | 5-6 |
| Information sources | 7 | PubMed, Embase, and Web of Science | 4 |
| Search | 8 | The PubMed, Embase and Web of Science databases were searched for papers published in English from January 1972 to February 2019, using basic search terms from combined text and Medica Subject Heading (MeSH) terms. These included a MeSH search using the term ‘Purpura, Schoenlein-Henoch’ and a keyword search using the term ‘Henoch-Schönlein purpura’, and terms related to unfavorable outcomes (including MeSH searches using the terms ‘Kidney Failure, Chronic’ and ‘Renal Insufficiency, Chronic’, and keyword searches using the term ‘end stage renal disease’ and ‘chronic renal disease’). This search strategy was adjusted to fit each database. The reference lists of relevant systematic reviews were also checked. | 5-6 |
| Study selection | 9 | Two reviewers independently screened the titles, abstracts, and full texts of retrieved articles based on pre-specified inclusion and exclusion criteria. Disagreements were resolved by a third reviewer. Cohort and case-control studies were included, whereas cross-sectional, case reports, review articles, comments, meeting abstracts, genetic association studies, and editorial comments were excluded. Data were independently extracted by two investigators, with any discrepancies resolved by a third investigator. Data collected included the characteristics of the studies (year of publication, country, and duration of follow-up), the demographic characteristics of the patients (e.g., numbers of patients and age), laboratory predictors, renal manifestations and renal histopathology at onset. | 6 |
| Data collection process | 10 | Data collected included the characteristics of the studies (year of publication, country, and duration of follow-up), the demographic characteristics of the patients (e.g., numbers of patients and age), laboratory predictors, renal manifestations and renal histopathology at onset. | 6 |
| Data items | 11 | Data collected included the characteristics of the studies (year of publication, country, and duration of follow-up), the demographic characteristics of the patients (e.g., numbers of patients and age), laboratory predictors, renal manifestations and renal histopathology at onset. | 6 |
| Risk of bias in individual studies | 12 | These papers are reported following the STROBE (Strengthening the Reporting of Observational Studies in Epidemiology) statement. Study quality was assessed using three main categories of the Newcastle-Ottawa scale. | 7 |
| Summary measures | 13 | We estimated the odds ratio (OR) with 95% confidence interval (CI) for dichotomous outcomes. A random-effects model was used regardless of heterogeneity. | 8 |
| Synthesis of results | 14 | Heterogeneity was assessed by subgroup analyses of follow-up durations(followed up for <5 or >5 years), ethnicity (Europe or Asia), date of publication (before 2000 or after 2000) and study quality across studies. Sensitivity analyses were conducted by removing each individual study from the overall analysis. If fewer than 10 studies were included, publication bias was not evaluated. All statistical analyses were performed using Stata 14.0 software (Stata Corp, College Station, TX, USA) | 8 |

Page 1 of 2

| **Section/topic** | **#** | **Checklist item** | **Reported on page #** |
| --- | --- | --- | --- |
| Risk of bias across studies | 15 | The control groups in the selected studies were not community-based. Because these studies were published between 1981 and 2017, there may have been a bias towards different treatment strategies. In addition, patients in three studies were followed up for less than 5 years, which may introduced bias. | 9-10 |
| Additional analyses | 16 | In those groups, followed up for >5 years, patients in Asia, publication after 2000, high quality studies, patients with initial renal features with nephrotic syndrome were associated with progression to poor outcomes. | 13 |
| **RESULTS** | | |  |
| Study selection | 17 | Initial screening identified 892 publications. Of these, only 9 case-control studies satisfied our inclusion criteria and were included in the meta-analysis | 8 |
| Study characteristics | 18 | These studies were published from January 1972 to February 2019. We obtained the full text and data of these studies. Of these 9 studies, two each in Finland and Turkey; one each in United Kingdom, Sweden, Japan, Poland and Germany. Thus, six studies were performed in Europe, three in Asia. The 9 studies included 969 patients with HSPN, with 160 experiencing unfavorable outcomes. The follow-up period ranged from 1.0 to 23.4 years, with the follow-up period in six studies being more than 5 years. | 8-9 |
| Risk of bias within studies | 19 | Seven studies were judged to be of high relative quality and two of medium quality, Because these studies were published between 1981 and 2017, there may have been a bias towards different treatment strategies. In addition, patients in three studies were followed up for less than 5 years, which may introduced bias. | 9-10 |
| Results of individual studies | 20 | The associations of baseline demographic and clinical characteristics (e.g., age, gender, and hypertension) and laboratory predictors (GFR, serum creatinine, plasma albumin, and level of proteinuria) of study subjects with prognosis in patients with HSPN were analyzed to assess risk factors for unfavorable outcomes. Older age (weighted mean difference [WMD] 1.77, 95% CI 0.35-3.18, p=0.014) and lower GFR level (WMD -23.93, 95% CI -33.78- -14.09, p=0.000) at onset were risk factors for unfavorable outcomes (Fig 2). By contrast, sex (male vs. female; OR 1.08, 95% CI 0.57-2.07, p=0.808) and hypertension (OR 1.80, 95% CI 0.60-5.38, p=0.292) at onset did not significantly affect patient outcomes. | 11 |
| Synthesis of results | 21 | older age, lower GFR, nephrotic syndrome, nephritic-nephrotic syndrome, and crescentic nephritis were associated with a poor prognosis. | 12-13 |
| Risk of bias across studies | 22 | In this meta-analysis, publication bias was not evaluated for fewer than 10 studies were included. | 14 |
| Additional analysis | 23 | In those groups, followed up for >5 years, patients in Asia, publication after 2000, high quality studies, patients with initial renal features with nephrotic syndrome were associated with progression to poor outcomes | 13 |
| **DISCUSSION** | | |  |
| Summary of evidence | 24 | The associations of baseline demographic and clinical characteristics (e.g., age, gender, and hypertension) and laboratory predictors (GFR, serum creatinine, plasma albumin, and level of proteinuria) of study subjects with prognosis in patients with HSPN were analyzed to assess risk factors for unfavorable outcomes. | 11 |
| Limitations | 25 | First, this analysis was based only on cohort and case-control studies, and the above risk factors cannot be relied on as causal factors associated with Henoch-Schönlein purpura nephritis progressing to unfavorable outcomes. Second, some of the risk factors studied, such as age, hypertension, proteinuria and GFR, were assessed in few publications, and differences in data presentation or details prevented a more robust meta-analyses of these factors, and some more recent articles could not be included in the analysis. Third, clinical and pathology features at baseline only are considered, without taking into account the treatment performed in the evaluation of the risk of progression over long term follow-up, may have been confounding factors in the included studies. Fourth, there were selection bias because of a small part literatures were exclusion, in that papers did not use Meadow's criteria. The wide range in publication dates, follow-up durations and ethnic populations, coupled with the development of new treatment technologies, may have been confounding factors in the included studies. Finally, few laboratory indicators were included, and it was impossible to comprehensively review all possible risk factors. | 18-19 |
| Conclusions | 26 | our results provide a detailed overview of factors associated with prognosis in children with HSPN. Older age, lower GFR, nephrotic syndrome, nephritic-nephrotic syndrome, and crescentic nephritis were associated with a poor prognosis. | 19 |
| **FUNDING** | | |  |
| Funding | 27 | None |  |

*From:*  Moher D, Liberati A, Tetzlaff J, Altman DG, The PRISMA Group (2009). Preferred Reporting Items for Systematic Reviews and Meta-Analyses: The PRISMA Statement. PLoS Med 6(7): e1000097. doi:10.1371/journal.pmed1000097

For more information, visit: **www.prisma-statement.org**.

Page 2 of 2
