# Supplementary figures and images for: Risk factors associated with IgA vasculitis with nephritis (Henoch–Schönlein purpura nephritis) progressing to unfavorable outcomes: A meta-analysis
Source: PLoS One. 2019 Oct 1;14(10):e0223218. doi: 10.1371/journal.pone.0223218 (PMC6772070; doi:10.1371/journal.pone.0223218)

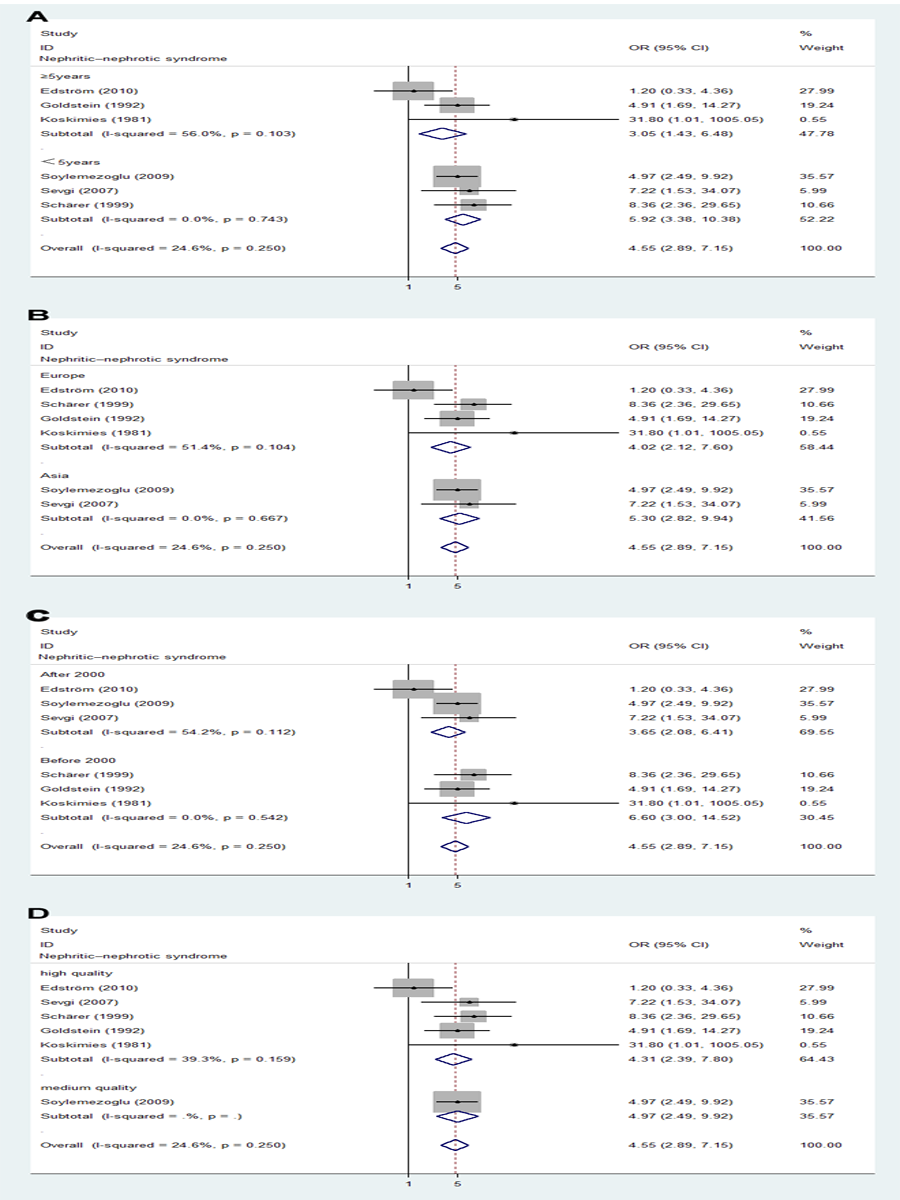

Supplement: S1 Fig — Subgroup analysis of in renal features with nephritic-nephrotic syndrome: (A) follow-up duration; (B) ethnicity; (C) date of publication; (D) study quality. (TIF) [file pone.0223218.s002.tif]

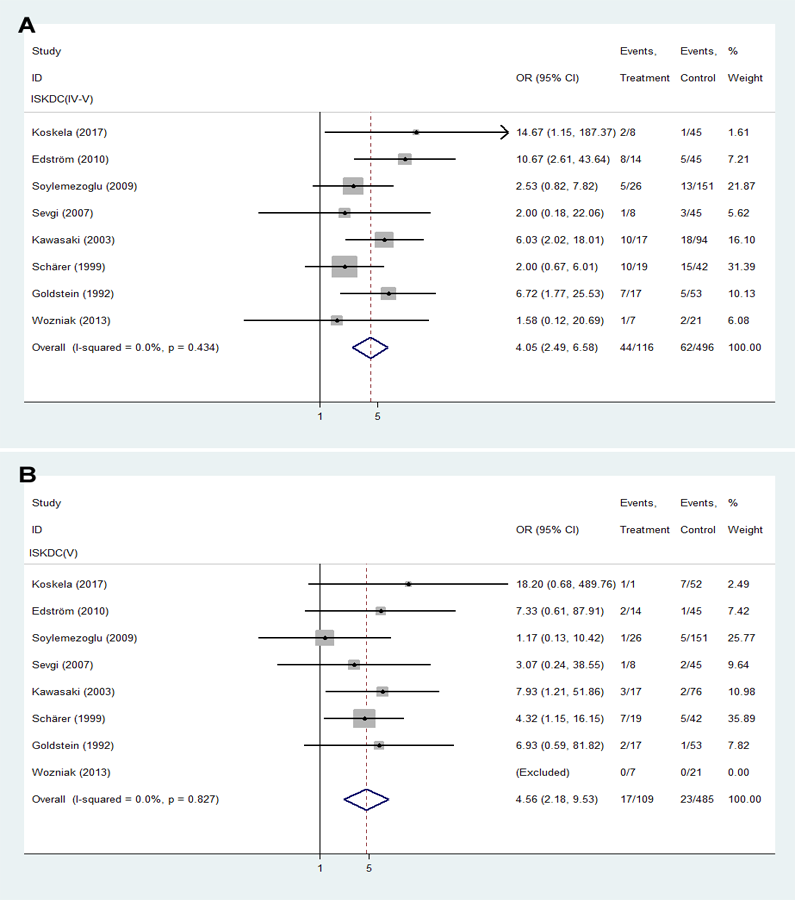

Supplement: S2 Fig — Forest plots of OR estimates for initial renal biopsy: (A) ISKDC grades IV-V; (B) ISKDC grades V. (TIF) [file pone.0223218.s003.tif]
