# Supplementary material for: Risk factors associated with IgA vasculitis with nephritis (Henoch–Schönlein purpura nephritis) progressing to unfavorable outcomes: A meta-analysis
Source: PLoS One. 2019 Oct 1;14(10):e0223218. doi: 10.1371/journal.pone.0223218 (PMC6772070; doi:10.1371/journal.pone.0223218)
Supplement: S1 Text — (DOCX) [file pone.0223218.s004.docx]

**The search strategy of Pubmed**

#1 "Purpura, Schoenlein-Henoch"[Mesh]

#2 Henoch-Schönlein purpura [All Fields]

#3 #1or#2

#4 "Kidney Failure, Chronic"[Mesh]

#5 end stage renal disease[All Fields]

#6 #4or#5

#7 "Renal Insufficiency, Chronic"[Mesh] OR "Kidney Failure, Chronic"[Mesh]

#8 chronic renal disease[All Fields]

#9 #7or#8

#10 #6or#9

#11 #3 and #10

**The search strategy of Embase**

#1 'anaphylactoid purpura'/exp

#2 'henoch-schönlein purpura’

#3 #1 or #2

#4 'end stage renal disease'/exp

#5 'end stage renal disease'

#6 #4 or #5

#7 'chronic renal disease'/exp

#8 'chronic renal disease’

#9 #7 or #8

#10 #6 or#9

#11 #3 and #10

**The search strategy of Web of science**

#1 ts=Henoch-Schönlein purpura

#2 ts=end stage renal disease

#3 ts=chronic renal disease

#4 #2 or #3

#5 #1 and #4
